# A Comparative Study of Koizumi and Longa Methods of Intraluminal Filament Middle Cerebral Artery Occlusion in Rats: Early Corticosterone and Inflammatory Response in the Hippocampus and Frontal Cortex

**DOI:** 10.3390/ijms222413544

**Published:** 2021-12-17

**Authors:** Mikhail V. Onufriev, Yulia V. Moiseeva, Marina Y. Zhanina, Natalia A. Lazareva, Natalia V. Gulyaeva

**Affiliations:** 1 Laboratory of Functional Biochemistry of the Nervous System, Institute of Higher Nervous Activity and Neurophysiology, Russian Academy of Sciences, 5A Butlerov Str., 117485 Moscow, Russia; mikeonuf@ihna.ru (M.V.O.); jumois@ihna.ru (Y.V.M.); m.u.kasatkina@gmail.com (M.Y.Z.); nalaza@rambler.ru (N.A.L.); 2 Research and Clinical Center for Neuropsychiatry of Moscow Healthcare Department, 43 Donskaya Str., 115419 Moscow, Russia

**Keywords:** stroke, middle cerebral artery occlusion, hippocampus, frontal cortex, distant damage, corticosterone, adrenocorticotropic hormone, interleukins, neuroinflammation

## Abstract

Two classical surgical approaches for intraluminal filament middle cerebral artery occlusion (MCAO), the Longa et al. (LM) and Koizumi et al. methods (KM), are used as alternatives in preclinical studies to induce stroke in rodents. Comparisons of these MCAO models in mice showed critical differences between them along with similarities (Smith et al. 2015; Morris et al. 2016). In this study, a direct comparison of MCAO-KM and MCAO-LM in rats was performed. Three days after MCAO, infarct volume, mortality rate, neurological deficit, and weight loss were similar in these models. MCAO-LM rats showed an increase in ACTH levels, while MCAO-KM rats demonstrated elevated corticosterone and interleukin-1β in blood serum. Corticosterone accumulation was detected in the frontal cortex (FC) and the hippocampus of the MCAO-KM group. IL1β beta increased in the ipsilateral hippocampus in the MCAO-KM group and decreased in the contralateral FC of MCAO-LM rats. Differences revealed between MCAO-KM and MCAO-LM suggest that corticosterone and interleukin-1β release as well as hippocampal accumulation is more expressed in MCAO-KM rats, predisposing them to corticosterone-dependent distant neuroinflammatory hippocampal damage. The differences between two models, particularly, malfunction of the hypothalamic–pituitary–adrenal axis, should be considered in the interpretation, comparison, and translation of pre-clinical experimental results.

## 1. Introduction

Ischemic stroke, a highly complicated and devastating neurological disease, is among the primary causes of death and long-term disability in the world [1]. The mechanisms of stroke pathogenesis, though numerous and multifaceted, have been vigorously studied for many decades, both experimental and in clinics. However, the therapy, though aimed at known key pathogenetic links, is not effective enough and the progress in treating stroke and its long-term sequelae has been frustratingly slow. Many essential details may remain obscure and this may be the main reason for this disappointment. For example, for many years, the penumbra tissue surrounding a brain infarct remains the main target for studies and neuroprotective strategies, though remote brain regions are injured as well [2].

Incomplete knowledge on stroke pathogenesis aspects prevents elaboration of valid experimental stroke models. This can explain why, over the past few decades, many neuroprotective drug candidates have shown promising results in preclinical rodent stroke models, though in clinical trials, these drugs have been unsuccessful, and none could show beneficial effects [3,4,5]. Lack of valid and suitable animal models for human stroke is regarded as a major reason for the translational failure and disappointment in developing effective neuroprotective drugs [6,7]. There is a vital need to rearrange experimental stroke setups and to use preclinical models more precisely mimicking the clinical features, risk factors, and comorbidities of stroke (stroke models on aged animals, animals with hypertension, diabetes, obesity, and hyperlipidemia) [4,8,9].

The impact of stroke on the major neurohumoral system of the organism, the hypothalamic–pituitary–adrenal (HPA) axis, is ignored in most popular rodent stroke models. Yet, the HPA system governs essential brain integrative control mechanisms, including stress responses and adaptations. Stress is one of the recognized key players in stroke pathogenesis, and the ability of a patient to respond to exteroceptive and enteroceptive stressors is critical for the onset, course, and outcome of stroke (see [10] for review). Stress-related triggering and deregulation of the HPA axis is manifested, in particular, in the release of excessive amounts of glucocorticoids, primarily cortisol in humans and corticosterone in rodents. The hippocampus, a brain region controlling both cognitive functions and emotional reactions, is selectively vulnerable to various stress factors, in particular, ischemia. The enrichment of the hippocampus with corticosteroid receptors may be a key link in mediating the detrimental effects of stress on this limbic structure inducing distant hippocampal damage. Stress-induced glucocorticoid-dependent hippocampal dysfunction is believed to be associated with the cognitive and emotional consequences of ischemic stroke [11]. The frontal cortex (FC) functions in close association with other regions of the brain participating in numerous aspects of learning and memory. The prefrontal cortex, the section of the FC at the very front of the brain, and the hippocampus have long been known to play a central role in various behavioral and cognitive functions [12,13]. Either directly or indirectly, neuronal projections from the hippocampus contribute to the hippocampal–prefrontal cortex circuit, which plays a critical role in cognitive and emotional regulation and memory consolidation.

Recently, we have shown in a preliminary experiment that two classical surgical approaches for modeling ischemic stroke, intraluminal filament middle cerebral artery occlusion (MCAO), following the Koizumi [14] (MCAO-KM) and Longa [15] methods (MCAO-LM), which are arbitrarily used worldwide in preclinical studies, may differ in cortisol response and hippocampal cortisol accumulation in rats [16]. The aim of this study was to perform a direct comparison of these two MCAO models. We assessed accumulation of cortisol and pro-inflammatory cytokines in the acute period after MCAO (3 days) in the hippocampus and frontal cortex and found important differences between the models, which are relevant for translation of the data received in pre-clinical studies using these MCAO models into clinical practice.

## 2. Results

### 2.1. Mortality, Neurological Deficits, and Brain Infarct Volume

**Figure 1:**
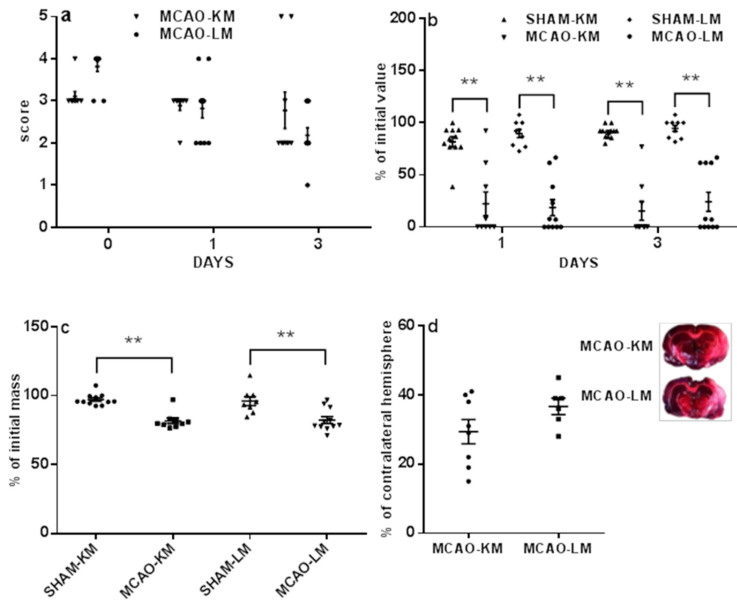
Neurological deficits (**a**,**b**), weight loss (**c**), and infarct volume (**d**) in MCAO-KM and MCAO-LM. (**a**) 5-score test after surgery (day 0), 1, and 3 days after surgery. (**b**) Tongue protrusion test 1 and 3 days after surgery; % of initial values. (**c**) Body mass 3 days after surgery; % of initial mass. (**d**) Infarct volume 3 days after surgery; % of the contralateral hemisphere; examples of TTC-stained frontal slices −4.5 ÷ −6.5 mm from bregma. ** *p* < 0.01 compared to the respective sham-operated groups.

The mortality in the MCAO-KM (17 survived of 30) and in MCAO-LM (17 survived of 27) did not differ (*p* = 0.42, Fisher exact test). The survived rats in both the MCAO-KM and MCAO-LM groups demonstrated dramatic neurological deficits 1 and 3 days after the surgery, both in the 5-score test (Figure 1a) and tongue protrusion test (Figure 1b). The neurological deficit according to the 5-score test (Figure 1a) decreased with time within 3 days (F(2,54) = 10.07, *p* = 0.0001); however, it did not depend on the model (F(1,54) = 0.006, *p* = 0.94; MCAO-K vs. MCAO-L on days 0, 1, and 3, *p* = 0.23, 1.0, and 0.41, respectively). The tongue protrusion test results (Figure 1b) showed a significant decrease (F(1,72) = 489.33, *p* = 0.0001; MCAO-KM vs. SHAM-KM and MCAO-LM vs. SHAM-LM on days 1 and 3, *p* = 0.0001) independently of the model (F(1,72) = 0.70, *p* = 0.40) and time (F(1.72) = 0.41, *p* = 0.52). The body mass loss on day 3 (Figure 1c) was significant in both MCAO groups (F(1,36) = 14.21, *p* = 0.00058; MCAO-K M vs. SHAM-KM and MCAO-LM vs. SHAM-LM, *p* = 0.0001), but it did not depend on the model either (F(1,36) = 0.79 *p* = 0.37; MCAO-KM vs. MCAO-LM, *p* = 0.98).

The infarct area usually included the frontoparietal and temporal parts of the neocortex as well as striatum. Infarct volume (Figure 1d) did not differ in MCAO-KM nor in MCAO-LM (31.33 ± 3.35 and 36.75 ± 2.35% of the healthy hemisphere, respectively; *p* = 0.23).

### 2.2. Corticosterone and Pro-Inflammatory Interleukins in Blood Serum

**Figure 2:**
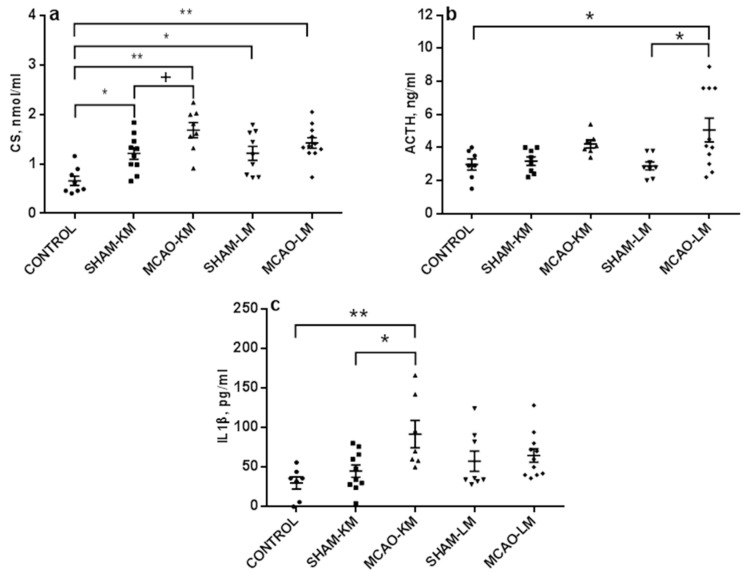
Blood levels of corticosterone (**a**), ACTH (**b**), and IL1β (**c**) in the MCAO-KM and MCAO-LM groups 3 days after surgery. (**a**) Corticosterone, nmol/mL. (**b**) ACTH, ng/mL. (**c**) IL1β, pg/mL. ** *p* < 0.01, * *p* < 0.05, and ^+^ *p* < 0.1 compared to the respective sham-operated groups or control rats.

Corticosterone level in blood serum on day 3 (Figure 2a) significantly changed after MCAO (F(1,34) = 7.10, *p* = 0.01). A trend to increase was evident in MCAO-KM only (MCAO vs. SHAM-KM, *p* = 0.07; MCAO-LM vs. SHAM-LM, *p* = 0.63). It should be noted that sham operation significantly augmented corticosterone in blood (Control vs. SHAM-KM, *p* = 0.026; Control vs. SHAM-LM, *p* = 0.030; Control vs. MCAO-KM, *p* = 0.0014; Control vs. MCAO-LM, *p* = 0.0008). The ACTH level in blood (Figure 2b) also showed a trend to increase (F(1,30) = 9.62, *p* = 0.004), though the increase was significant in MCAO-LM only (MCAO-KM vs. SHAM-KM, *p* = 0.51; MCAO-LM vs. SHAM-LM, *p* = 0.02). Sham operation did not affect the ACTH level in either MCAO model.

Changes in the IL1β levels in blood serum were similar to those of corticosterone: the IL1β level (Figure 2c) increased (F(1,32) = 5.76, *p* = 0.022), but the augmentation was significant in MCAO-KM rats only (MCAO-KM vs. SHAM-KM, *p* = 0.035; MCAO-LM vs. SHAM-LM, *p* = 0.96). In the MCAO-KM model, the difference between the control (intact) animals and MCAO group was also significant (*p* = 0.006), and the difference between the models remained significant when the MCAO values were expressed in % of respective sham-operated animals (MCAO-KM 203.81 ± 38.21 and MCAO-LM 112.57 ± 14.81; *p* = 0.02). The levels of IL6 and TNFα in blood (Figure S1) did not change after MCAO in either MCAO model (IL6: F(1,35) = 0.02, *p* = 0,88; TNFα: F(1,32) = 1.62, *p* = 0.21).

### 2.3. Corticosterone and Pro-Inflammatory Interleukins in the Hippocampus and Frontal Cortex

**Figure 3:**
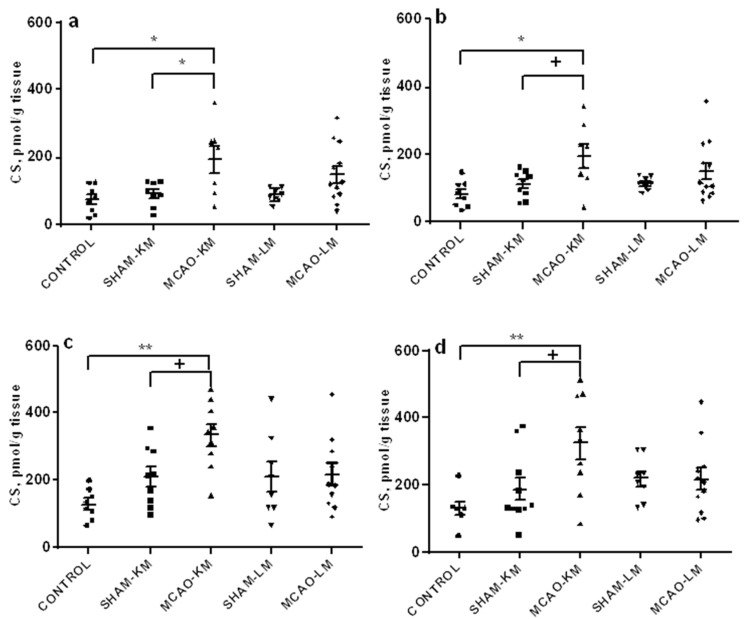
Corticosterone levels in the hippocampus (**a**,**b**) and FC (**c**,**d**) in MCAO-KM and MCAO-LM groups 3 days after surgery. (**a**) Ipsilateral hippocampus. (**b**) Contralateral hippocampus. (**c**) Ipsilateral FC. (**d**) Contralateral FC. (**a**–**d**) Corticosterone, pmol/g tissue.** *p* < 0.01, * *p* < 0.05, and ^+^ *p* < 0.1 compared to the respective sham-operated groups or control rats.

The corticosterone level in the ipsilateral hippocampus (Figure 3a) increased 3 days after MCAO (F(1,31) = 10.57, *p* = 0.027), though only MCAO-KM showed a significant trend to increase (MCAO-KM vs. SHAM-KM, *p* = 0.050; MCAO-LM vs. SHAM-L, *p* = 0.26). Corticosterone in the ipsilateral hippocampus of MCAO-KM rats was also higher than in the control animals (*p* = 0.048). In the contralateral hippocampus, the trend of a corticosterone increase was seen in the MCAO-KM rats (F(1,33) = 6.45, *p* = 0.015; MCAO-KM vs. SHAM-KM, *p* = 0.091; MCAO-LM vs. SHAM-LM, *p* = 0.67). Corticosterone in the ipsilateral hippocampus of MCAO-KM rats was also higher than in the controls (*p* = 0.011). In the ipsilateral FC (Figure 3b), corticosterone showed a trend to increase (F(1,33) = 3.52, *p* = 0.069), and this was evident only in MCAO-KM rats (MCAO-KM vs. SHAM-KM, *p* = 0.075; MCAO-LM vs. SHAM-LM, *p* = 0.99; MCAO-KM vs. MCAO-LM, *p* = 0.079). MCAO-KM rats showed higher corticosterone than the controls (intact rats) in the ipsilateral FC (*p* = 0.0008), and the difference between models was significant when MCAO values were expressed in % of respective sham-operated animals (MCAO-KM 158.89 ± 15.67; MCAO-LM 102.90 ± 14.90, *p* = 0.019). In the contralateral FC, corticosterone changed similarly, showing a trend to increase (F(1,34) = 3.47; *p* = 0.07), and this was evident only in MCAO-KM rats (MCAO-KM vs. MCAO-LM, *p* = 0.079; MCAO-LM vs. SHAM-LM, *p* = 0.99). MCAO-KM rats showed higher corticosterone than the control rats (*p* = 0.006), and the difference between models was significant when the MCAO values were expressed in % of respective sham-operated animals (MCAO-KM 172.97 ± 26.05 and MCAO-LM 99.16 ± 14.87, *p* = 0.019).

**Figure 4:**
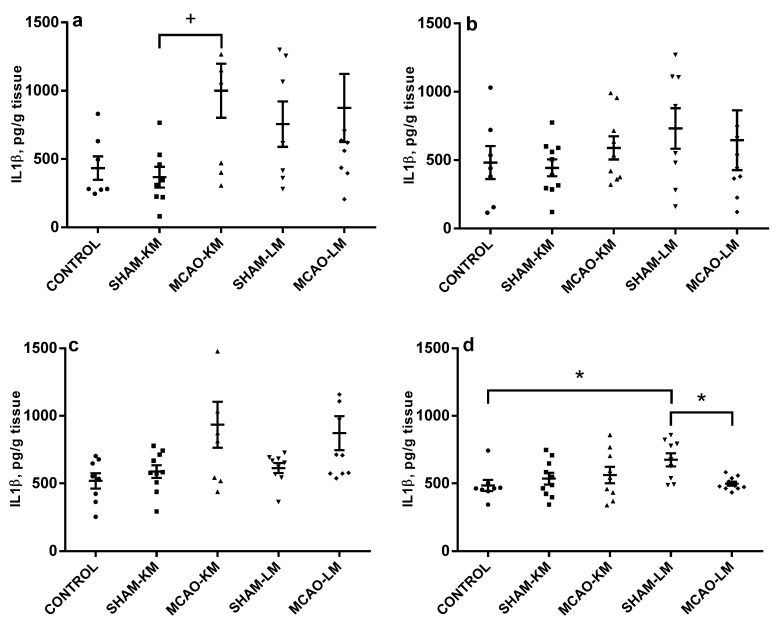
IL1β levels in the hippocampus (**a**,**b**) and FC (**c**,**d**) in MCAO-KM and MCAO-LM groups 3 days after surgery. (**a**) Ipsilateral hippocampus. (**b**) Contralateral hippocampus. (**c**) Ipsilateral FC. (**d**) Contralateral FC. (**a**–**d**) IL1β, pg/g tissue. * *p* < 0.05 and ^+^ *p* < 0.1 compared to the respective sham-operated groups or control rats.

An augmentation in IL1β level was evident in the ipsilateral hippocampus (F(1,27) = 4.23, *p* = 0.049) and FC (F(1,33) = 8.31, *p* = 0.006) (Figure 4). This was a result of the significant trend of IL1β to increase in MCAO-KM animals (ipsilateral hippocampus: MCAO-KM vs. SHAM-KM, *p* = 0.090; MCAO-LM vs. SHAM-LM, *p* = 0.97; ipsilateral FC: MCAO-KM vs. SHAM-KM, *p* = 0.10; MCAO-LM vs. SHAM-LM, *p* = 0.31). In the contralateral hippocampus and FC, no changes in IL1β could be detected (hippocampus: F(1,32) = 0.046, *p* = 0.82; FC: F(1,34) = 2.88, *p* = 0.10). In the contralateral FC, an IL1β decrease was revealed in MCAO-LM rats (MCAO-LM vs. SHAM-LM, *p* = 0.036), and the difference between models was significant when the MCAO values were expressed in % of the respective sham-operated animals (MCAO-KM 114.39 ± 12.37 and MCAO-LM 77.58 ± 2.35, *p* = 0.006).

The IL6 levels did not change after MCAO, irrespective of the type of surgery either in the hippocampus or in the FC in ipsilateral and contralateral hemispheres (F(1,34) = 0.01, *p* = 0.91; F(1,35) = 1.1, *p* = 0.30; F(1,34) = 0.39, *p* = 0.53; F(1,35) = 0.36, *p* = 0.54) (Figure S2). No major TNFα changes could be detected after MCAO either (hippocampus: F(1,35) = 1.10, *p* = 0.29; FC: F(1,32) = 0.75, *p* = 0.39); the only exception was a decrease in TNFα in the ipsilateral FC as compared to the sham group in MCAO-LM rats (Figure S3).

## 3. Discussion

Two classical surgical approaches for intraluminal filament, MCAO-KM and MCAO-LM, are used as alternatives in preclinical studies to induce stroke in rodents. Comparisons of these MCAO models in mice [17,18] showed critical differences between them along with similarities. In this study, a direct comparison of MCAO-KM and MCAO-LM in rats was performed. Three days after MCAO, infarct volume, mortality rate, neurological deficits, and weight loss were similar in these models. MCAO-LM rats showed an increase in ACTH levels in blood serum 3 days after the surgery, while MCAO-KM rats demonstrated increases in blood corticosterone and IL1β. Corticosterone accumulation was detected in the FC and hippocampus of the MCAO-KM group. IL1β beta increased in the ipsilateral hippocampus of the MCAO-KM group and decreased in the contralateral FC of MCAO-LM rats. Differences between MCAO-KM and MCAO-LM suggest that corticosterone and IL1β release as well as hippocampal accumulation are more expressed in MCAO-KM rats and thus that MCAO-KM predisposes them to corticosterone-dependent distant neuroinflammatory hippocampal damage.

### 3.1. HPA Axis, Stress Response, and Brain Ischemia

The HPA axis, a key system realizing stress response, underlies both acute stress response and long-term adaptation. Glucocorticoids (cortisol in humans, corticosterone in rodents) released from the adrenal cortex during stress responses are key messengers in the integrative regulation of brain adaptive plasticity. Endocrine alterations of the HPA axis are among essential stress-induced alterations after cerebral ischemia. Activation of the HPA axis is one of the first physiological responses to cerebral ischemia occurring in the first hours after ischemia and leading to a prolonged increase in the concentration of glucocorticoids in the blood [19,20,21]. The concept of the tight interlacing of stroke with stress load is commonly recognized, the connections between stress and stroke being numerous and intricate. The association of ischemic stroke with stress response is proven by lots of studies in patients and animal models, and functioning of the HPA, the major neurohumoral system, mediates this association (see [10] for review). Hypoxia-ischemia in rodents is accompanied by increased activity of the HPA axis and abundant secretion of corticosterone, which could potentially exacerbate brain damage via activation of receptors of glucocorticoids and increase neuronal vulnerability. This is of primary translational relevance since a systematic review and analysis of a number of clinical ischemic stroke studies suggest that stress response assessed as HPA axis activation is among the most promising prognostic biomarkers [22]. It is assumed that the deregulation of the HPA system and predisposition to stress-induced mental illnesses, including depression, are based on a violation of the glucocorticoid regulation of the feedback system, as well as an imbalance between central corticosteroid receptors [23,24]. The modulation of the HPA axis activity is attributed, in particular, to the hippocampus [25,26], and includes signaling through mineralocorticoid and glucocorticoid receptors, their high level of expression being observed in the hippocampal subfields CA1, CA2, CA3, and the dentate gyrus [27].

### 3.2. HPA Axis, Brain Ischemia, and Neuroinflammation

HPA axis dysfunction is closely associated with pro-inflammatory events [11]. In acute ischemic stroke patients, serum IL6 predicts cortisol release, suggesting that ischemia-induced IL6 release in blood modulates HPA axis [28]. Remarkably, Slowik et al. (2002) [29] suggested that prognostic significance of hypercortisolemia in acute ischemic stroke patients is related to the inflammatory response rather than to the stress response. In experimental models of ischemic stroke, HPA system activity is associated with neuroinflammation, mediated by increased expression of pro-inflammatory cytokines TNFα, IL1β, and IL6. These cytokines can modulate the size of ischemic damage in experimental stroke in rodents [30,31]. These pro-inflammatory cytokines, and especially IL1β, are able to activate the HPA axis and cause an increase in the level of corticosterone in the systemic circulation [32,33]. Increased levels of these cytokines were found in cerebrospinal fluid and blood of patients after ischemic stroke in [34,35,36]. In a MCAO model of stroke, acute stress was shown to increase brain ischemic damage, the effect of stress being mediated at least partially by pro-inflammatory cytokines IL1β and TNFα [37,38].

### 3.3. Ischemic Stroke and Distant Hippocampal Damage

Stroke, a severe neurological pathology characterized by high mortality, is often followed by the development of post-stroke depressive disorders and cognitive impairments. Recently, numerous data have been accumulated, indicating that the damage after a stroke is not limited to the infarction area only, but also spreads to non-ischemized regions of the brain, causing secondary damage that, very frequently, is remote and delayed. After focal damage to the cerebral cortex and/or striatum, secondary changes are observed in areas of the brain remote from the infarction zone, primarily in the hippocampus [39,40]. Though acute brain damage caused by ischemic stroke in the middle cerebral artery circulation primarily affects the cerebral cortex and striatum, the key mechanisms of dementia and depression are associated with hippocampal dysfunction. The hippocampus controls both cognitive and emotional functions. The above reasons brought about a new hypothesis on the distant hippocampal damage as a key link in the pathogenesis of cognitive and psychiatric disturbances after focal brain injury, including delayed consequences of ischemic stroke [2,11]. According to this hypothesis, an excess of glucocorticoids secreted after a focal brain damage, in particular in patients with abnormal stress-responses due to HPA axis malfunction, interacts with receptors of glucocorticoids in the hippocampus, inducing signaling pathways that stimulate neuroinflammation and subsequent events, including hippocampal neurodegeneration and disturbances in neurogenesis. Thus, functional and structural damage to the hippocampus, a brain region selectively vulnerable to external factors and responding to them by increased cytokine secretion, most possibly forms a basis for cognitive function disturbances and psychopathology development. Besides cognitive and emotional post-stroke disturbances, stroke may trigger epileptogenesis, often comorbid with depression. Common molecular and cellular mechanisms of these comorbidities include distant hippocampal damage associated with HPA axis dysfunction, the malfunction of glucocorticoid receptors, and development of neuroinflammation, leading to neurodegeneration and loss of hippocampal neurons as well as formation of aberrant neural networks [41]. Since the hippocampus is a place of life-long neurogenesis, glucocorticoid-mediated mechanisms of hippocampal damage are involved in alterations in subgranular neurogenesis and contribute to both cognitive and emotional disturbances and to epileptogenesis as well [42].

This concept still should be confirmed by more pre-clinical and clinical data, though associations between brain ischemia and hippocampal damage have been described by several groups in rodent experiments. The adrenal stress hormones glucocorticoids acting through their receptors abundant in the hippocampus are critical to the physiological control of different executive functions and behavioral responses, which usually represent adaptive reactions. Signal transduction mediated by hippocampal receptors of glucocorticoids regulates the genomic activities underlying neuroplasticity and behavioral adjustment to stressogenic factors. Milot et al. [43] demonstrated increased sensitization and responsiveness of the HPA system at long intervals after cerebral ischemia in rodents. These effects contributed to post-ischemic cognitive impairments (disturbances in spatial memory) and hippocampal degeneration. Kadar et al. [44] reviewed numerous earlier studies, using five different experimental models in rats (normal aging, hypoxia, prolonged corticosterone administration, brain ischemia, and cholinesterase inhibition), showing that cognitive dysfunction is invariably accompanied by hippocampal CA1 and CA3 pyramidal cells degeneration, though the most affected area depended on the specific model used. Rami et al. [45] focused on the synergy between chronic corticosterone treatment and cerebral ischemia in producing damage to hippocampal neurons. Their data support the hypothesis that corticosterone treatment accompanied by an ischemic insult cause an extended increase in neuronal [Ca2+], hippocampal noncalbindinergic neurons being particularly prone to ischemic insults. Using a MCAO model of ischemic stroke, Onufriev et al. [46] showed that accumulation of corticosterone in the hippocampus was associated with an increase in the proinflammatory cytokine IL1β. Importantly, in our present study, trends to increased corticosterone accumulation demonstrated for the hippocampus were evident in the FC, too. Hippocampus and prefrontal cortex are central structures of the cortico-limbic network involved in higher cognitive processes, including learning and memory, as well as emotional regulation [47]. Since the hippocampus and prefrontal cortex play a central role in different behavioral and cognitive functions, their dysconnectivity may underlie various brain diseases, including dementia and depression [48]. Corticoid-dependent dysfunction of FC, in addition to the hippocampus, may essentially contribute to delayed post-stroke brain disturbances. Accumulation of corticosterone in the hippocampus and FC not only in the damaged, but also in the healthy hemisphere, confirms the concept of secondary distant glucocorticoid-dependent damage after ischemic stroke.

### 3.4. Rodent MCAO Models Are Not Identical

MCAO is believed to belong to most clinically important surgical models of ischemic stroke. Among a selection of potential methodological approaches, the most common involves insertion of a monofilament to occlude the artery. Two classical surgical approaches for intraluminal filament MCAO, the Koizumi [14] and Longa [15] methods, are used worldwide for ischemic stroke modeling in preclinical studies. By default, most scientists using either of these models regard them as very similar, if not equivalent, and evaluate and compare the data obtained by different groups from this perspective. However, this approach does not seem appropriate. MCAO-KM involves insertion of a monofilament via the common carotid artery, while MCAO-LM via the external carotid artery. A direct comparison and detailed evaluation of these models in mice [17,18] showed both similarities and critical differences between them. Although Morris et al. [18] found no significant difference in total lesion volume and survival rates between MCAO-KM and MCAO-LM, the latter model produced significantly greater reperfusion in mice. Smith et al. [17] found that the MCAO-LM method in mice produced a greater and more robust inflammatory response versus the MCAO-KM method, suggesting that MCAO-LM is superior for the study of both short- and long-term outcomes of ischemic stroke.

In this study, we have compared MCAO-KM and MCAO-LM in rats since these models, similarly to respective murine MCAO models, are currently used arbitrarily in various laboratories worldwide. The comparison of the two MCAO models in rats within 3 days after the surgery performed in our study gives evidence that their simultaneous use can help to explore an essential, though still underestimated and understudied, aspect of ischemic stroke-activation of the HPA axis and related distant hippocampal damage. MCAO-LM mimics ischemic stroke with lesser HPA axis activation, while MCAO-KM mimics stroke-induced activation of the HPA axis with potential distant damage to the hippocampus and FC and higher risk of delayed cognitive and emotional disturbances. Comparative studies at longer time points are needed as well as behavioral and histological studies in these MCAO models aimed at revealing cognitive and emotional disturbances and hippocampal damage.

The importance of control groups in MCAO studies deserves a special attention. As a rule, sham-operated rats are used in preclinical stroke studies, since they are regarded as more adequate groups for comparison with stroke animals than intact controls. Though this approach may be the best of available ones, it does not seem perfect. Using sham-operated controls implies that changes and consequences introduced by surgery (surgical stress) can be easily subtracted, and a simple difference gives the net effect of experimental stroke. Unfortunately, it is impossible to prove this statement. Sham surgery is stressogenic; however, simple arithmetic rules do not work in the physiology of higher organisms, and the contribution of different stress factors into the general effects seen in a stroke group can be barely regarded as just additive. The interaction between myriads of shared and distinct mechanisms at different levels induced by multiple acting factors cannot be predicted. The physiological effects of handling and even sham injection can exert effects exceeding those of the “main factor” studied (see examples [49,50]). Analysis of miRNAs confirms that sham operation may have independent effects [51]. The above rationale clearly shows that no ideal control to MCAO exists, and the more different controls, the better understanding of the MCAO effect we can provide. Therefore, control animals without manipulation, in addition to the respective shams, should be used. Taking this into account, we performed multiple comparisons (MCAO vs. sham, MCAO vs. intact, sham vs. intact, MCAO-LM vs. MCAO-KM, and comparisons between MCAO as a percentage of sham) since changes in the sham-operated groups may either mask real alterations in MCAO animals or provide false ones. An example illustrating the necessity of using different control groups can be demonstrated by analyzing the data in Figure 4d. In the contralateral FC, an IL1β decrease was revealed in MCAO-LM rats; however, this decrease is obviously associated with an apparent increase in the SHAM-LM group. A similar situation may cause the appearance of significant difference in TNFα in the ipsilateral FC in MCAO-LM rats (Figure 3c).

## 4. Materials and Methods

### 4.1. Environment and Housing

The study was performed on 85 male 3-month-old Wistar rats (BW 200–300 g; average 256.5 g). The animals were purchased from the “Stolbovaya” Breeding Center (Moscow Region, Russia) and were housed in acrylic cages with spruce shavings provided as bedding, in the institutional vivarium. The rats were kept in a 12 h:12 h day cycle, with free access to food and water. All experiments with animals were performed in accordance with the EU Directive 2010/63/EU. The experimental protocol was approved by the Ethical Commission of the Institute of Higher Nervous Activity and Neurophysiology, Russian Academy of Sciences (protocol number 10, 10 December 2012). All efforts were made to minimize animal suffering.

The rats were randomly assigned to 5 groups: middle cerebral artery occlusion (MCAO) performed using the Koizumi method (MCAO-KM); sham-operated Koizumi method (SHAM-KM); MCAO performed using the Longa method (MCAO-LM); sham-operated Longa method (SHAM-LM); and intact control (CONT). At the beginning of the experiment, the groups included 17, 11, 13, 9, and 8 rats, respectively. An additional 27 rats were used for infarct volume assessment in MCAO-KM and MCAO-LM rats; at the beginning of the experiment, the groups included 13 and 14 rats, respectively.

### 4.2. Modeling Ischemic Stroke

MCAO-KM. The surgery was performed according to the method of Koizumi et al. [14]. Rats were anesthetized with isoflurane. An incision was made in the neck area and, pushing the muscle tissue on the left side, penetration to the common carotid artery was performed and ligatures applied to it, as well as to the external and internal carotid arteries. A nylon filament (3–0) with a rounded end was inserted through the hole at the bifurcation site onto the external and internal branches and advanced along the internal carotid artery to the middle cerebral artery. Then the ligature on the internal carotid artery was tightened to fix the filament. The occlusion lasted for 60 min; common, external, and internal carotid arteries remained ligated, and the body temperature of the animal was maintained at 37 ± 0.5 °C. Then, the filament was removed and the ligature on the internal carotid artery was tightened. Thus, after occlusion, the common, external, and internal carotid arteries on the left side remained ligated and reperfusion occurred mainly at the expense of the Willis circle. In the SHAM-KM group, all these manipulations were performed, except for the introduction of the filament.

MCAO-LM. The surgery was performed according to the method proposed by Longa et al. [15] under isoflurane anesthesia. Through an incision in the neck of the animal, the common carotid and external carotid arteries were assessed and ligated. After electrocoagulation and dissection of a fragment of the left external carotid artery near the bifurcation, a filament was inserted through the remaining part of the artery and pushed through the internal carotid artery to the intersection with the middle cerebral artery. The occlusion lasted for 60 min, while the body temperature of the rat was maintained in the range of 37 ± 0.5 °C. Then, the filament was extracted, and blood flow restored along the ipsilateral common carotid artery, which was released from the ligature after the extraction of the filament. In the SHAM-LM group, all these manipulations were performed, except of the introduction of the filament.

### 4.3. Assessment of Neurological Deficits

1. Five-point neurological scale. Motor and behavioral changes were assessed using a 0–5 point grading scale [52] at 1 h following MCA occlusion, and daily prior to sacrifice. This standard test used conventionally to assess the efficiency of focal brain ischemia in rodents, is based on a 5-point behavioral scale and allows to evaluate the functional state of the contralateral foreleg of the rat, the presence of turns and circulation in the contralateral side, as well as the mobility of the animal: 0, no deficit; 1. failure to extend right forepaw fully; 2. decreased grip of right forelimb while tail pulled; 3. spontaneous circling or walking to contralateral side; 4. walks only when stimulated with depressed level of consciousness; 5. unresponsive to stimulation.
2. Tongue protrusion test. This test indicates the ability of the rat to lick peanut butter out from a thin full glass cylinder left in the cage overnight [53]. The length of the butter pile, from the beginning of the cylinder to the level of the remaining butter, shows the ability of the animal to protrude its tongue, which is impaired during the acute phase of MCAO.

### 4.4. Collection of Biomaterial

Blood from the caudal vein was collected under brief isoflurane anesthesia before MCAO and 72 h after MCAO (Day 3). On Day 3, the animals were instantly decapitated, and the following biological material was obtained: post-decapitation blood (serum); and the ipsi- and contralateral regions of the brain: the hippocampus and frontal cortex (FC, +6.1–3.0 mm from bregma). Brain regions were homogenized using a Potter homogenizer in a 10-fold excess of a cold buffer for homogenization (0.1% NP-40, a protease inhibitor (Roche), and phosphate-buffered saline (PBS)) with 10 impacts of the pestle at a rotation speed of 1000 rpm. The homogenate was centrifuged to obtain a soluble fraction of proteins (supernatant), which was aliquoted and stored at −80 °C before biochemical studies.

### 4.5. Enzyme-Linked Immunosorbent Assays (ELISA)

To determine the serum and brain tissue corticosterone levels, kits for the enzyme-linked immunosorbent assay (Kit Corticosterone for 96 tests, cat. no. EIA4164; DRG) were used; the kits allow to detect both free and transport protein-bound corticosterone by a competitive ELISA method.

The levels of proinflammatory cytokines IL1β, IL6, and TNFα in blood serum and brain tissue of rats were measured by R&D Systems Quantikine ELISA Kits according to the manufacturer’s instructions (cat.no. SRLB00; cat. No. SR6000B; cat. No. SRTA00).

### 4.6. Statistical Analysis

The data were checked for normality using the Shapiro–Wilk W-test. For statistical analysis of the data on neurological deficits after MCAO assessed by the 5-point scale and tongue protrusion test, repeated measures ANOVA was used followed by post-hoc Tukey HSD tests. For the analysis of biochemical data, factorial ANOVA followed by post-hoc Tukey HSD tests were used. To compare the parameters in the experimental groups with those in intact animals, one-way ANOVA with Tukey HSD tests were used. For an additional comparison of two MCAO models, the percentage of change in % was calculated as the ratio of the value in a MCAO group to an average of respective sham group (MCAO*100%/SHAM) and compared using *t*-tests. Difference in the infarction volume was assessed using *t*-tests. The data on rat mortality after MCAO were analyzed using Chi-square tests. *p* values <0.05 and <0.1, *p* > 0.05 were considered to indicate a significant difference or statistical trend, respectively. The results are presented as individual points and the mean ± SEM.

## 5. Conclusions

A successful translation of basic insights into medical practice depends, in particular, on the ability of researchers to use the results of clinical trials and refine the validity of experimental models. Although the involvement of augmented cortisol in ischemic stroke outcome and delayed post-stroke brain disturbances are recognized based on results of clinical studies, this knowledge and respective hypotheses did not affect the preclinical studies. The involvement of HPA axis activation and related hippocampal dysfunction should be understood to bridge the gap between stroke pathogenesis and treatment and to ensure proper clinical translation of experimental results. Models of ischemic stress reproducing the state of the HPA axis in clinical cases are needed, and this should be considered when planning the preclinical research. The differences between the two MCAO models, particularly related to HPA axis function, should be considered in the interpretation, comparison, and translation of experimental results. These results have considerable implications on stroke model selection for researchers.

## Data Availability

Data available on the demand.

## Supplementary Materials

The following materials are available online at https://www.mdpi.com/article/10.3390/ijms222413544/s1.

## Abbreviations

FC	frontal cortex
HPA	hypothalamic–pituitary–adrenal
MCAO	middle cerebral artery occlusion
MCAO-KM	middle cerebral artery occlusion, Koizumi et al. model
MCAO-LM	middle cerebral artery occlusion, Longa et al. model
ELISA	enzyme-linked immunosorbent assay
IL1β	interleukin-1beta
IL6	interleukin-6
TNFα	tumor necrosis factor-alpha
