# Supplementary material for: A Comparative Study of Koizumi and Longa Methods of Intraluminal Filament Middle Cerebral Artery Occlusion in Rats: Early Corticosterone and Inflammatory Response in the Hippocampus and Frontal Cortex
Source: Int J Mol Sci. 2021 Dec 17;22(24):13544. doi: 10.3390/ijms222413544 (PMC8703333; doi:10.3390/ijms222413544)
Supplement: Supplementary file 1 [file ijms-22-13544-s001.zip › ijms-1497714-supplementary.pdf]

Supplementary figures to the manuscript "A Comparative Study of Koizumi and Longa Methods of Intraluminal Filament Middle Cerebral Artery Occlusion in Rats: Early Corticosterone and Inflammatory Response in the Hippocampus and Frontal Cortex" by Mikhail V. Onufriev, Yulia V. Moiseeva, Marina A. Zhanina, Natalia A. Lazareva, Natalia V. Gulyaeva

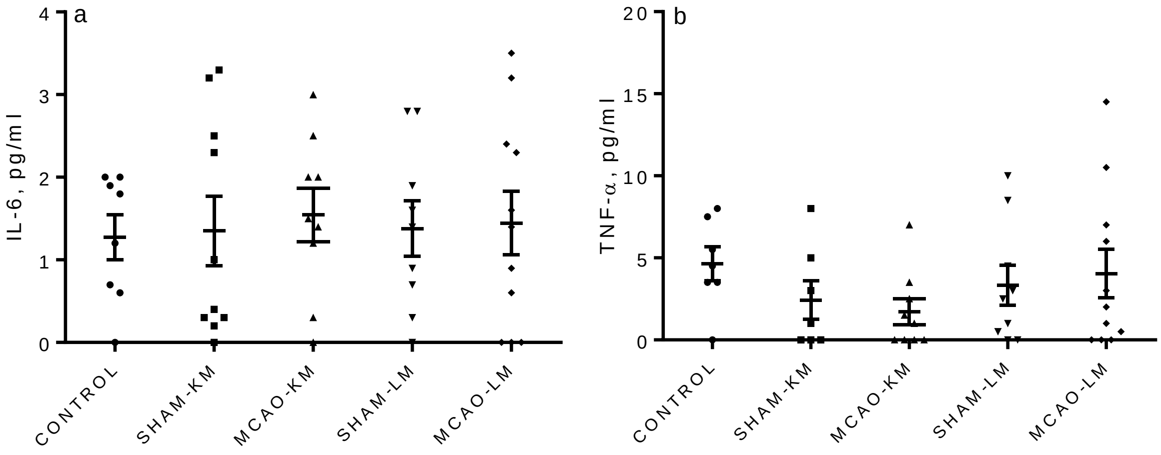

Figure S1. Blood levels of IL6 (a) and TNFα (b) in MCAO-KM and MCAO-LM groups 3 days after surgery. a, IL6, pg/ml. b, TNFα, pg/ml. No significant differences between groups have been detected.

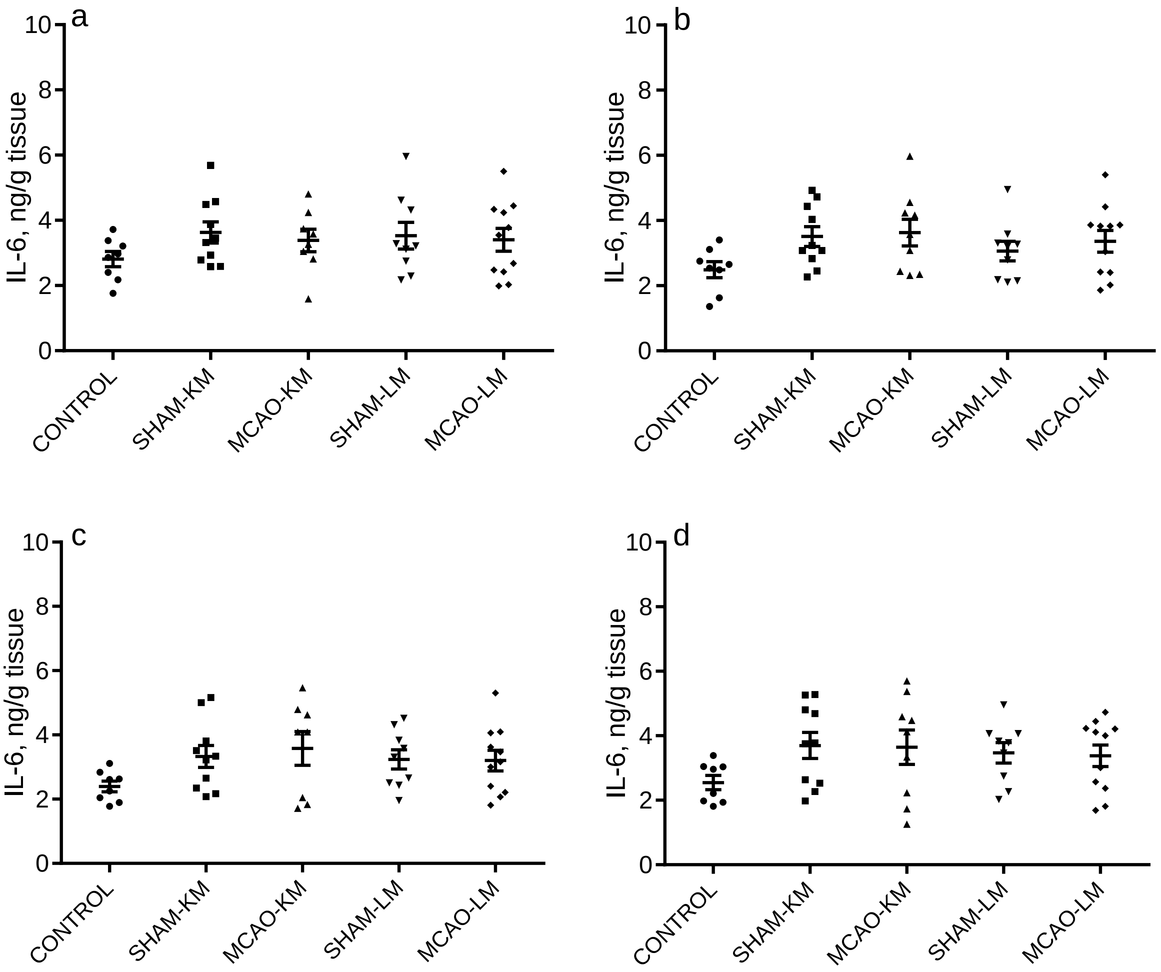

Figure S2. IL6 levels in the hippocampus (a, b) and FC (c, d) in MCAO-KM and MCAO-LM groups 3 days after surgery. a, ipsilateral hippocampus. b, contralateral hippocampus. c, ipsilateral FC. d, contralateral FC. a-d, IL6, ng/g tissue. No significant differences between groups have been detected.

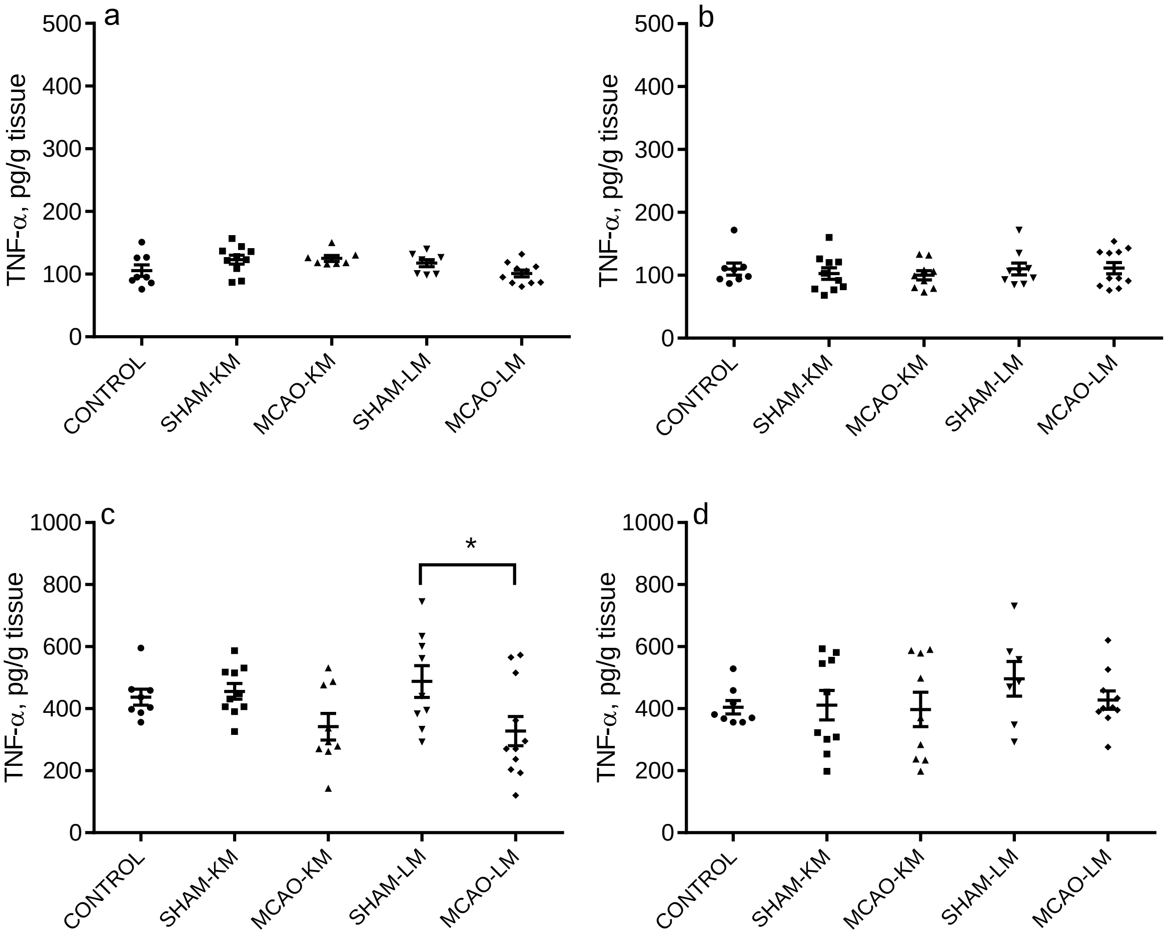

Figure S3. TNFα levels in the hippocampus (a, b) and FC (c, d) in MCAO-KM and MCAO-LM groups 3 days after surgery. a, ipsilateral hippocampus. b, contralateral hippocampus. c, ipsilateral FC. d, contralateral FC. a-d, TNFα, ng/g tissue. *- $p < 0.05$, from respective sham operated group.
